# Personal values, marketing attitudes and nutrition trust are associated with patronage of convenience food outlets in the Asia-Pacific region: a cross-sectional study

**DOI:** 10.1186/s41043-017-0082-4

**Published:** 2017-02-16

**Authors:** Breanna De Jong, Anthony Worsley, Wei Chun Wang, Rani Sarmugam, Quynh Pham, Judhiastuty Februhartanty, Stacey Ridley

**Affiliations:** 1 School of Exercise and Nutrition Sciences, Deakin University, Burwood, Australia; 2 Peninsula Clinical School, Faculty of Medicine, Nursing and Health Sciences, Monash University, Clayton, Australia; 3 Health Promotion Board, Singapore, Singapore; 4 SEAMEO REFCON (Regional Centre for Food and Nutrition) Universitas Indonesia, Jakarta, Indonesia; 5 Behavioural Nutrition, Institute for Physical Activity and Nutrition Research, School of Exercise and Nutrition Sciences, Deakin University, 221 Burwood Highway, Burwood, Victoria 3125 Australia

**Keywords:** Asia-Pacific, Attitudes, Convenience food outlets, Nutrition trust, Personal segmentation, Survey, Values

## Abstract

**Background:**

An online cross-sectional survey examined the relationships between the demographic characteristics, personal values, trust in sources of nutrition information and the use of convenience food outlets among middle-class household food providers in the Asia-Pacific region.

**Methods:**

The survey was administered to 3945 household food providers in Melbourne, Singapore, Shanghai, Vietnam and Indonesia in late 2013. Information about demographics, personal values, trust in sources of nutrition information and use of convenience food outlets was elicited. Exploratory factor analysis, two-step clustering and logistic regression were employed.

**Results:**

The analyses found that the use of convenience food outlets was positively related to hedonist values and trust in food industry sources of nutrition information. However, lesser use of convenience food outlets and trust in health sources of nutrition information was associated with traditional (community-oriented) values.

**Conclusions:**

Further replication and extension of these findings would be useful. However, they suggest that improvements in the quality of foods sold in convenience food outlets combined with stronger regulation of food marketing and long-term food education are required.

## Background

High prevalence of overweight and obesity was once considered to be a problem experienced only in high-income countries such as Australia and Singapore [1], but prevalence rates are now increasing rapidly in low- and middle-income countries (LMIC) including Indonesia, Vietnam and China [2, 3]. For example, between 1991 and 2011, the prevalence of obesity in China increased from 2.9 to 11.8% among men and from 4.6 to 11.0% among women [3]. Bhurosy and Jeewon [4] suggest that not only high-income countries but also LMICs are increasing their intakes of energy dense, nutrient poor (EDNP) foods, such as confectionery, cakes, biscuits and sugar-sweetened beverages. These dietary changes, along with decreases in physical activity levels, have led to increases in overweight and obesity [5].

These changes are also known to be key risk factors for obesity [3] and non-communicable diseases (NCD) [5]. These are the leading causes of death globally, accounting for over 68% of deaths worldwide in 2012 [1]. In the past, NCDs mainly affected higher-income countries, but now almost three quarters of all NCD deaths (28 million per annum) occur in LMICs [1].

One explanation for this is the rise in the number of middle-income families within developing nations. The middle class is defined as those lying between the 20th and 80th global income percentile, who earn between US$10 and $100 a day [6]. The number of people considered middle-class worldwide is expected to reach 4.9 billion by 2030, with Asia having the fastest growing middle-class population worldwide [6]. One key trend observed among these emerging middle-class populations is the change in dietary patterns [7]. This consists of a switch from traditional diets (legumes, pulses, vegetables) to those that include more meat and EDNP products, similar to that of many western diets [8].

Asian consumers have always largely relied on wet markets as places to source their food, but with the rise in middle-income families and a more western-style of living, supermarkets and convenience food outlets are rapidly becoming major food retailers in these countries [9, 10]. Such stores are significant sources of EDNP foods and beverages [7]. They include fast food restaurants (McDonalds, KFC), vending machines, street stalls, newsagents, petrol stations and other similar outlets. The number of convenience food outlets appears to be greater in lower socio-economic communities than in more affluent communities [11, 12]. However, little research has been conducted on the use of convenience food outlets in LMICs such as Vietnam, Indonesia and China.

Food now goes through many different hands and travels long distances. One consequence of this long supply chain is that consumers are separated from, and ignorant of, many aspects of the food supply including the ways foods are grown, processed, distributed and marketed [12, 13]. Additionally, in the Asia-Pacific region, there has been a large increase in the marketing and advertising of EDNP products [10, 12, 14]. Marketers influence consumers by appealing to their psychological or ‘psychogenic’ needs which underpin daily behaviours. These include interacting needs including the needs for social recognition, maximisation of pleasure and the avoidance of pain, stability or coherence in daily life and the enhancement of self-esteem [15]. For example, classical Coca Cola advertising portrayed social success and sexual attraction. These industry sources offer food and nutrition information which often competes with information provided by health organisations and practitioners, and families and friends, making it difficult for consumers to know which sources to trust [16, 17].

The main aim of this paper was to examine the families’ use of convenience food outlets, which are major sources of EDNP products. Further, we wanted to examine the associations of several factors, described below, with convenience food outlet use. Briefly, we proposed the following hypotheses.

- *Demographic differences:* Previous research has shown that food companies target families with children (pester power) and that women and older people are more interested in food and health issues than others [8]. Therefore, we expected that people with children would be more likely to trust industry sources of nutrition information and to use convenience food outlets than other people, and conversely women and older people would be less likely to trust industry sources and to patronise convenience food outlets less often than others. We also took the opportunity to test the expectation that people who purchase foods often from convenience outlets might have higher body mass indices (BMI) than other people, based on the assumption that these outlets predominantly sell energy dense products.
- *Personal values*: These are defined as guiding principles in people’s lives. Schwartz [18] values taxonomy postulates ten sets of values that can be subsumed into three broad categories: Self-transcendent values such as Equality-nature, the importance of caring for others, for the community, animals and the environment; Tradition-security, valuing tradition and various forms of security; and Hedonism, placing an emphasis on excitement and pleasure in life. We hypothesised that Hedonists may be more predisposed to the exciting imagery and promises made in food marketing and so would be more likely to trust industry sources of information and to use convenience outlets that sell processed foods and beverages. In contrast, we expected those with high self-transcendent and Tradition-security values would be less likely to trust industry information and less likely to use convenience food outlets.
- *Trust in sources of nutrition information:* In previous work, we have shown that trust in sources of nutrition information falls into three groups, industry sources (e.g. food advertising), health sources (e.g. advice from doctors) and social sources (friends and families) [19]. Since the products sold by convenience food outlets are largely EDNP products, we expected that those who trust industry nutrition information would be more likely to use convenience food outlets than those who trust health or social sources.
- *Attitudes to food marketing:* We expected that people who have positive attitudes to food marketing will be more likely to trust industry information and to patronise convenience food outlets.
- *Economic development and indices of consumerism:* We expected that residents of developed economies (Singapore and Melbourne) would be less likely to trust industry information and hold more negative attitudes to food marketing than those in developing economies (Indonesia, Vietnam and Shanghai) because of their longer experience with the lack of veracity of some forms of food marketing [20].

## Methods

### Design and sampling

This study is based on an online survey administered between December 2013 and January 2014 by the Global Market Insite (GMI), a global market research company. GMI invited potential respondents from the company’s database of registered adults living in Melbourne, Australia; Shanghai, China; Indonesia; Singapore and Vietnam to participate through a link to the survey. Adequate representation of gender, age and consumer groups was ensured by quota procedures. In total, 3951 people participated in the survey. Of these, 770 were from Melbourne, Australia, 807 were from Shanghai, China, 793 were from Indonesia, 771 were from Singapore and 810 were from Vietnam. The main inclusion criteria required participants to be household food providers and over 18 years of age, and a quota of approximately 40:60 male/female ratios was set.

### Procedure

To ensure people from diverse cultural backgrounds could understand the questions, the survey questionnaire (titled ‘*International Families and Food study*’) was translated into Vietnamese, Bahasa Indonesia and Chinese by GMI. The translations were then checked by nationals of these countries (JF, QP, WC). GMI then invited potential respondents to take part in the survey via their database, until the quota for each stratum was met. Ethics permission for the study was granted by the Deakin University Health Ethics Advisory Group (HEAG 2013-163). Informed consent was implied through completion of the survey.

### The questionnaire

The questionnaire included eight broad sections, but only the sections described below are relevant to this study. Further information about the findings from this survey are to be found in Worsley et al. 2016a, 2016b [21, 22] and in a series of reports: https://blogs.deakin.edu.au/apfnc/. Many of the items used in this exploratory study were derived from discussions between the authors as well as from unpublished studies and the previous research of the authors.

#### Food shopping

Respondents were asked: *During the past month how often have you bought a food or a beverage from…*(then followed a list of four retail food outlets, eight of which were convenience food stores, shown in Table 1). Five-point rating scales were used to indicate these frequencies: *no (1), once or twice (2), yes, several times (3), every day (4), several times a day (5)*. The list of convenience food outlets was derived from discussion among the authors.

**Table 1:** Demographic and body weight characteristics of the respondents

	Melbourne *n* = 769	Shanghai *n* = 807	Indonesia *n* = 788	Singapore *n* = 771	Vietnam *n* = 810	Total *n* = 3945
Gender: female (%)	58.4	57.2	59.6	49.3	60.5	57.1
Age: mean (std dev)	41.43 (12.70)	37.81 (10.53)	32.97 (9.07)	37.45 (11.68)	29.25 (7.35)	35.72 (11.23)
Marital status: married/de facto (%)	60.9	77.4	56.9	55.3	51.4	60.4
Education: bachelor’s degree or higher (%)	58.8	89.5	80.7	74.2	89.6	78.8
Families with children between 0 and 5 years (%)	20.4	34.9	39.3	25.9	54.2	35.2
Families with children between 6 and 12 (%)	18.1	16.5	30.9	21.4	27.1	23
Families with children between 13 and 18 (%)	15.6	14.7	25.1	19.2	21.4	19.2
BMI mean (std dev)	26.89 (7.03)	23.60 (6.92)	23.11 (5.17)	23.08 (4.53)	20.81 (3.32)	23.47 (5.91)

Several other sets of questions were also asked, as described below. The items were generally measured on 5-point agreement response scales, e.g. strongly disagree (1) to strongly agree (5). Confirmatory factor analyses (principal components with varimax rotation) were used to derive internally reliable scales; details are available from the corresponding author. As noted in the “Background” section, these constructs were hypothesised to impact respondents’ patronage of convenience stores. They included the following:

#### Trust in sources of nutrition information

Respondents were presented with a list of 12 sources of nutrition information, derived from our earlier work [20], and were asked to rate how much they trusted each of them using 5-point rating scales; low trust (1) to high trust (5). A subsequent principal component analysis with varimax rotation of the trust ratings derived three factors, which were provisionally named: *Industry Sources* (food industry associations, food labels, dieting websites, food manufacturers, soft drink manufacturers, food retailers, Cronbach’s *α* = 0.85), *Health Sources* (medical doctors, health organisations [e.g. Heart Foundation], dieticians/nutritionists, government health departments, traditional healers [naturopaths, herbalists], Cronbach’s *α* = 0.79) and *Social Sources* (adult family members, friends, Cronbach’s *α* = 0.78). Scores were calculated for each respondent on each factor for use in the regression analyses below.

#### Perceived importance of food knowledge and skills (Cronbach’s *α* = 0.90)

This was based on eight items about the nutrient content, processing, preparation and environmental impact of foods [23].

#### Attitudes to food marketing

*Support for unhealthy food promotion* (Cronbach’s *α* = 0.90), e.g. promotion of confectionary and soft drinks in supermarkets, vending machines, schools and in advertising.

#### Attitudes to food marketing

Support for *healthy food promotion* (Cronbach’s *α* = 0.73), e.g. the marketing of fruit and vegetables, water, milk and dairy products.

#### Personal values

Three scales were derived from the 23-item Portrait of Values inventory [18] and named as follows: Security-tradition-conformity (Cronbach’s *α* = 0.68), Hedonism (*α* = 0.73) and Equality-nature (*α* = 0.74; similar to Schwartz’s self-transcendent values, universalism values) [18].

#### Demographic characteristics

Details were elicited of the respondents’ age, gender and marital status (coding: single/widowed/separated/divorced = 1, married/cohabiting = 2). The number of children residing in the household, if any, was recoded into three age bands: 0 to 5, 6 to 12 and 13 to 18 years. In addition, self-reported height and weight were recorded and converted into body mass indices (BMI; kg/m^2^). BMI was standardised with a mean of 0 and standard deviation of 1. Educational level was assessed as university educated or not university educated.

### Data analysis

SPSS version 22 was used for all the analyses. Descriptive statistics were calculated to summarise the respondents’ demographic characteristics. The use of convenience stores in the five cities/countries is shown in Table 1. The usage ratings for the eight convenience stores were input into a two-step cluster analysis (two-step program, SPSS). This program identified two groups of respondents according to their usage of convenience stores: cluster 1 high users and cluster 2 low users. Then, logistic regression analysis was used to compare high and low users of convenience food outlets across the demographic, country of residence, personal values, attitudes to marketing and trust in sources of nutrition information characteristics of the respondents (Table 3).

## Results

### The demographic characteristics of the sample

Fifty-seven percent of the respondents were female; the mean age was 35.72 (SD 11.23) years. Sixty percent were married or cohabiting, whilst 25.7% of respondents were considered overweight. Over one third (35.2%) of households had a child under 5 years, 23% had a child aged 6–12 years and 19.2% cared for a child aged 13 to 18 years of age. Mean BMI for the whole sample based on self-reported height and weight was 23.47 (SD 5.91); it ranged from 20.81 (3.32) in Vietnam to 26.89 (7.03) in Melbourne. As expected from our sampling strategy, most respondents had undergone some form of higher education (78.8%). Further details are available from the corresponding author.

### Convenience outlets usage

There were statistically significant differences in the usage of convenience food outlets between the five countries (Table 2). Inspection of Table 2 shows that fewer Melbournians and Indonesians reported using convenience food outlets and in contrast Vietnamese followed by Shanghainese respondents patronised them most frequently. Overall, the most popular convenience outlets were convenience stores (e.g. 7-eleven), street stalls or small shops and fast food restaurants (e.g. McDonalds, KFC) (Table 2).

**Table 2:** Reported frequent patronage of convenience food outlets

	%Melbourne *n* = 770	%Shanghai *n* = 807	%Indonesia *n* = 793	%Singapore *n* = 771	%Vietnam *n* = 810	%Total *n* = 3951	Chi sq.
Convenience store (7-eleven)	3.4	11.0	9.5	5.8	11.5	8.3	553.1
Street stall or small shop	3.8	7.1	5.8	7.4	15.7	8.0	382.9
Fast food restaurant (e.g. McDonalds, KFC)	3.5	7.1	4.0	6.9	8.1	5.9	50.4
Cinema or theatre	3.2	6.6	2.1	4.8	9.4	5.3	129.9
Sport venue	2.9	7.7	3.3	4.5	10.7	5.9	133.2
Petrol station	2.2	7.1	3.7	5.2	10.1	5.7	96.4
Vending machine	2.7	6.9	1.8	6.5	8.5	5.3	109.5
Newsagent	2.9	8.4	3.0	5.8	10.7	6.2	166.0

Note: All country comparisons were statistically significant (*p* < 0.001). The chi-square tests compared infrequent use (ratings 1 and 2) and weekly use (rating 3) with frequent use (ratings 4 and 5)

Question: During the past month how often have you bought a food or a beverage from… ? (Five-point rating scales: No (1), once or twice (2), yes several times a week (3), everyday (4), several times a day (5). The percentages of frequent use of stores (ratings 4 + 5) are shown in this table

*The cluster analysis* showed that respondents in cluster 1 were less likely to purchase food and beverages from the eight convenience venues than respondents grouped in cluster 2 (Table 3). Cluster 1 includes respondents who use convenience less often (infrequent users) than those in cluster 2 (frequent users).

**Table 3:** Results of the cluster analysis of convenience food outlet use

Venue	Cluster 1 (*N* = 2715) low-use mean	Cluster 2 (*N* = 1236) high-use mean	Importance (%)
Sports centre	1.20	2.69	1.00
Cinema or theatre	1.35	2.73	1.00
Vending machine	1.19	2.62	1.00
Street stall or small shop	1.70	2.9	1.00
Newsagent	1.13	2.68	1.00
Petrol station	1.23	2.64	1.00
Cafe	1.81	2.94	1.00
A fast food restaurant (KFC, McDonalds)	1.76	2.83	0.99
A convenience store (7-eleven)	1.84	2.99	0.94

The logistic regression analysis showed that respondents in Shanghai, Vietnam, Indonesia and Singapore relied on convenience food outlets more than Melbournians (Table 4). Furthermore, respondents who placed importance on food knowledge and skills and the promotion of healthy food were less likely to use convenience food outlets, as were people who were older and trusted health sources of nutrition information. In comparison, respondents who held hedonist values and who trusted industry sources of nutrition information were more likely to use convenience outlets, as were women and those who had children between the ages of 0–5 and 13–18 years.

**Table 4:** Major associations with the use of convenience food outlets

Variable	Cluster 1 High b(*n* = 1236) Mean (sd)	Cluster 2 Low (*n* = 2715) Mean (sd)	B	S.E.	Wald	Sig	Exp (B)	95% CI for Exp (B)	
								Lower	Upper
Shanghai vs. Melbourne	.25 (.44)	.18 (.39)	−1.000	.156	48.804	.000	.333	.244	.453
Vietnam vs. Melbourne	.28 (.45)	.17 (.38)	−.971	.161	36.476	.000	.379	.276	.519
Indonesia vs. Melbourne	.18 (.38)	.21 (.41)	−.754	.158	22.678	.000	.470	.345	.642
Singapore vs. Melbourne	.19 (.40)	.20 (.40)	−.632	.153	16.971	.000	.531	.393	.718
Sex (% female)	48.5	61.0	.407	.084	23.450	.000	1.502	1.274	1.771
Age (years)	32.05 (8.9)	37.37 (11.8)	−.031	.005	36.114	.000	.969	.959	.979
Marital status (%married)	59.1	61.2	−.072	0.104	0.475	.491	0.931	0.759	1.142
Children 0–5 years (*n*)	.66 (.87)	.39 (.71)	.344	.060	32.907	.000	1.410	1.254	1.586
Children 6–12 years (*n*)	.36 (.92)	.61 (.01)	.030	.069	0.189	.664	1.030	0.900	1.180
Children 13–18 years (*n*)	.29 (.73)	.22 (.56)	.204	.070	8.354	.004	1.226	1.068	1.407
Standardised BMI	−.10 (.96)	.05 (1.01)	.004	.044	0.010	.922	1.004	0.921	1.095
Security-tradition-conformity values	3.62 (.65)	3.61 (.64)	−.092	.082	1.241	.265	0.912	0.777	1.072
Hedonism values	3.61 (.65)	3.17 (.72)	.951	.077	151.36	.000	2.587	2.224	3.011
Equality-nature values	3.81 (.64)	3.91 (.61)	−.232	.091	6.539	.011	0.793	0.664	0.947
Perceived importance of food skills and knowledge	3.85 (.69)	4.12 (.63)	−.600	.078	59.129	.000	.549	.471	0.640
Support for healthy food promotion	3.94 (.74)	4.18 (.65)	−.174	.068	6.613	.010	0.840	0.736	0.959
Support for unhealthy food promotion	3.03 (.81)	2.48 (.78)	.426	0.057	56.204	.000	1.531	1.369	1.711
Trust in health sources of nutrition information	3.78 (.77)	3.94 (.66)	−.333	.071	22.148	.000	0.717	0.624	0.823
Trust in industry sources of nutrition information	3.14 (.74)	2.81 (.67)	.613	.069	77.810	.000	1.845	1.611	2.115
Trust in social sources of nutrition information	3.68 (.70)	3.68 (.77)	.021	.066	0.105	.746	1.021	0.898	1.162
Constant			1.865	.648	8.277	.004	6.459		

Note: This table compares high users of convenience stores with low users. The columns headed Clusters 1 and 2 show the bivariate differences between the clusters in the means and standard deviations (or percentages) of the independent variables

## Discussion

Most of our hypotheses were supported, with higher users of convenience food outlets indicating more positive views of industry and marketing, whilst also being more hedonistic than those who used them less frequently. In contrast, lower users held stronger community-oriented values, distrusted food marketing and industry but were more likely to trust health and social sources of nutrition information.

Generally, the demographic associations were smaller than the associations with country of residence, personal values, attitudes and trust. However, the gender and age associations were consistent with earlier findings relating to health concerns [24], attitudes and practices [8] and trust [25].

The lack of an association between BMI and convenience outlet use highlights the complexity of the influences on population body weight. A recent systematic review found few associations between BMI and physical environments [26]. The zero association we found suggests the need for careful investigation of products sold by convenience outlets, customers’ purchasing practices, and in particular, their dietary practices.

The logistic regression findings (Table 3) show, in particular, that hedonist values, support for unhealthy food promotion, country of residence (especially Shanghai vs. Melbourne) and the perceived importance of *skills and knowledge* were major associates of convenience food outlet use. The country-of-residence differences are consistent with our hypothesis that stage of economic development is likely to influence the adoption of consumerism. The values and attitude findings, in turn, suggest that people’s worldviews may markedly influence the adoption of consumerism.

The positive relationships observed between the presence of children (13–18, 0–5 years) and use of convenience food outlets is consistent with past research which has shown that children are targeted by the food industry, especially through their interactive, attractive advertising [27, 28]. The lack of a similar association among households with 6–12-year-old children may reflect either less intensive marketing pressure on this age group or greater parental efforts to promote healthier foods in this age group. This requires further investigation.

Contrary to our expectation that men would be greater users of these outlets (hypothesis a), we found that women were more likely to be high users. This may be a result of women generally being the main household food providers who face increased demands and time pressure associated with child care and employment in paid work outside the home [29], resulting in less time for food preparation and more reliance on convenience foods and their outlets.

Overall, the findings are consistent with the changes in living practices that are occurring in developing countries, including more women working in paid employment outside the home [29] and smaller households, resulting in less time for food preparation. This provides processed food companies with opportunities to market their products in ways that particularly attract hedonistic household food providers [7]. The ways in which they do this have been described and criticised [10], but it is clear that they have strong support from some sections of these societies, particularly government economic development ministries [7].

### Implications for nutrition promotion

The findings have several implications for health and nutrition promotion. There are at least two groups of household food providers who have different interests, attitudes and values. They require different forms of communication and support from public health authorities. First, the finding that hedonist food providers are more likely to trust industry sources of nutrition information suggests that they may be more vulnerable than others to food industry marketing tactics [22]. Hedonist people tend to be drawn to exciting, innovative advertisements (see Coca Cola advertisements), which is a common strategy used by the food industry in order to engage consumers [10]. Health authorities should consider use of similar approaches to this group, emphasising fun and sociability associated with healthy foods and beverages. This will require considerable funding and organisation and collaboration with primary industries, small businesses and community groups who are able to provide these products. For example, vending machines are capable of providing healthy foods and beverages. The strong negative relationship of the perceived importance of *skills and knowledge* suggests important roles for schools and community education agencies since the more knowledge and skills people have, the more they will be able to prepare meals within prevailing time and resource constraints.

In other analyses of these data (not shown here, available from the corresponding author), personal values, trust and attitudes to marketing were shown to mediate the relationships between household demographic characteristics and convenience food outlets. This suggests a second approach. These mediating variables could be altered by health communicators to decrease trust in industry sources, and increase trust in health sources, and so decrease the use of convenience food outlets. This would require financial resources from cash-strapped health authorities and could have negative effects on many small businesses. A more feasible approach would be a combination of improvements in the quality of foods sold in convenience food outlets combined with stronger regulation of food marketing and long-term food education to create higher expectations about food quality in the population.

### Strengths and future implications

The research had a number of strengths, two of which are particularly important. First, the study is the first of its kind to reach a broad range of middle-class household food providers, residing five countries within the Asia-Pacific region. Second, the online administration of the survey allowed the respondents to provide large amounts of information about their food habits and attitudes in a relatively inexpensive manner.

Because these findings are new in the area of global health and nutrition, future research should aim to replicate them in order to examine further ways how personal values and attitudes to food provision can influence householders’ decisions to purchase food and beverages from convenience food outlets.

### Limitations

There were several limitations to this research. First, quota sampling was used to recruit middle-class samples from the five localities. Whilst random probability sampling has been considered to be superior to quota sampling, online quota sampling is more likely to yield more representative samples than random probability sampling [30]. Nevertheless, there is a clear need in future studies to examine the views and experiences of households from lower SES backgrounds, since they also experience the nutrition transition and various aspects of consumerism. Second, qualitative approaches are required to examine the present findings more deeply. Third, future studies should extend the present approach to the examination of household dietary practices. These studies should use more detailed objective measures of convenience food outlet patronage to ascertain whether frequent users have less healthy dietary practices and higher body weights than less frequent users.

## Conclusions

The findings show that personal values, country of residence, attitudes to food marketing, trust in sources of nutrition information, perceived importance of food skills and knowledge and householder demographic characteristics are important associations of the patronage of convenience food outlets. Two distinct groups of household food providers exist in the Asia-Pacific region (1) those who hold hedonist values, trust industry sources of information self-transcendent values and (2) those who trust health sources and distrust industry sources of information and use convenience food outlets less frequently.

Further replication and extension of this study is required so that healthy food promotion strategies can be developed to meet the needs of households in the Asia-Pacific region.

## Abbreviations

BMI: Body mass indices
EDNP: Energy dense, nutrient poor
GMI: Global Market Insite
LMIC: Low- and middle-income countries
NCD: Non-communicable diseases
